# In Vitro Comparison of Gingival Epithesis Materials: Color Stability, Surface Properties, and Microbial Adhesion After Staining

**DOI:** 10.3390/dj14030142

**Published:** 2026-03-04

**Authors:** Ellen Pick, Andrea Gubler, Thomas Attin, Patrick R. Schmidlin

**Affiliations:** Clinic of Conservative and Preventive Dentistry, Center for Dental Medicine, University of Zurich, 8032 Zurich, Switzerland; ellen.pick@zzm.uzh.ch (E.P.); andrea.gubler@zzm.uzh.ch (A.G.); thomas.attin@zzm.uzh.ch (T.A.)

**Keywords:** epitheses, silicon-based materials, nylon-based material, high-performance polymer, acrylic resin, discoloration, roughness, scanning electron microscopy, biofilm

## Abstract

**Background**: This in vitro study compared color stability, surface properties, and microbial adhesion of four gingival epithesis materials (silicone: Gingivamoll^®^; nylon: Valplast^®^; PETG-based high-performance polymer: Eldy Plus^®^; PMMA: Palapress^®^) after staining. **Methods**: Standardized specimens (10 × 10 × 2 mm; n = 18/material) underwent 15 or 30 staining cycles (sequential immersion in coffee, curry, tea, and 40% alcohol). Color (CIELAB) and color difference (ΔE00), gloss (G), and surface roughness (Ra) were measured at baseline and after 15 and 30 cycles; surface morphology was assessed by SEM. Microbial adhesion was assessed using a six-species biofilm model and quantified as log CFU at baseline and after 15 and 30 cycles. **Results**: All materials showed clinically relevant discoloration (ΔE00 > 2). Valplast^®^ exhibited the greatest color change (*p* < 0.05), while color change in other materials remained lower. Gingivamoll^®^ showed the lowest gloss and highest roughness, whereas other materials remained smoother; roughness increased significantly over time (*p* < 0.05). SEM revealed a coating on the hard materials and nodular agglomerates on silicone. Biofilm CFU did not differ over time or between materials (all *p* > 0.05). **Conclusions**: Staining induced material-dependent changes in color and surface properties, with Valplast^®^ most prone to discoloration and silicone showing high roughness and nodular surface changes, contrasting with coatings on hard materials. Microbial adhesion analysis yielded pilot-level results, intended to inform the design of future investigations.

## 1. Introduction

The esthetic rehabilitation of patients with periodontitis and severe periodontal attachment loss remains challenging and often results in gingival recessions and interdental “black triangles,” which can compromise both the perceived outcome and the predictability of available treatment options [1].

In addition to conventional periodontal-prosthetic interventions, gingival epitheses offer a non-invasive alternative that may be cost-effective and esthetically acceptable for selected patients. These removable prostheses are intended to replace lost gingival contours—most commonly in the maxillary anterior region—and can be fabricated from soft materials (e.g., silicones) or from harder materials such as modified acrylic resins, nylon-based materials, or PETG-based high-performance polymers [2,3,4].

Silicone-based materials are typically more flexible and fracture-resistant, but they may be more prone to discoloration over time in the oral environment [5,6]. Acrylic resins, widely used for dentures, are generally more color-stable yet comparatively brittle [7]. CAD–CAM-milled PETG-based polymers have recently been described as flexible and comfortable; however, they may offer limited color choices. Nylon-based materials are also considered flexible but—similarly to silicones—have been associated with color instability and plaque accumulation over time [3,8]. Despite the distinct advantages and limitations of these materials, there is currently no consensus among clinicians and dental technicians regarding the optimal choice for gingival epitheses [9].

Color stability and surface properties are critical parameters for gingival epithesis materials, as these prostheses are continuously exposed in the esthetic zone and subjected to dietary staining agents. Previous studies on dental and prosthetic polymers have demonstrated that staining may not only impair optical properties but also induce surface degradation and promote bacterial colonization [10,11]. However, comparative data on the behavior of commonly used gingival epithesis materials under simulated oral exposure remain limited.

Therefore, the null hypothesis of this study was that no significant differences exist among the investigated gingival epithesis materials regarding color stability, surface properties, or microbial adhesion after simulated oral exposure and staining.

## 2. Materials and Methods

### 2.1. Material Selection and Sample Fabrication

Four commonly used materials for gingival epithesis fabrication were compared: a silicone-based material (Gingivamoll^®^, Detax GmbH & Co. KG, Ettlingen, Germany), a nylon-based material (Valplast^®^, Johannes Weithas dental-kunststoffe GmbH & Co. KG, Lütjenburg, Germany), an acrylic resin (Palapress^®^ pink, Kulzer GmbH, Hanau, Germany), and a high-performance polymer (Eldy Plus^®^, Dental Plus GmbH, Samerberg, Germany). For each material, 18 specimens with standardized dimensions (10 × 10 × 2 mm) were fabricated.

Gingivamoll^®^ and Palapress^®^ specimens were prepared according to the manufacturers’ instructions. The silicone material was packed into a flask and heat-cured, whereas the acrylic resin was fabricated using a powder–liquid system, mixed and poured into a mold, and subsequently heat-polymerized [12,13]. Valplast^®^ specimens were fabricated by a dental laboratory (Baumgartner & Studer AG, Zurich, Switzerland) following the manufacturer’s reference guide [14]. Eldy Plus^®^ is a medical-grade, high-performance polymer based on glycol-modified polyethylene terephthalate (PETG) and was provided as pre-polymerized blocks in pink opal [15]. These blocks were then sectioned to the standardized sample dimensions using a low-speed saw (ISOMET^®^, Buehler Ltd., Lake Bluff, IL, USA).

To obtain comparably smooth surfaces, all specimens were polished using silicon carbide waterproof abrasive paper with increasing FEPA grit sizes (P1200, P2000, and P4000) under constant water cooling. Each specimen was polished for 60 s on each grit, then stored in a sealed container filled with water for 1 h.

### 2.2. Exposure Cycles

Each specimen underwent an initial assessment to establish baseline values for color, gloss, and surface roughness. Measurements were performed immediately after fabrication and after 1 h of water immersion. All specimens per material were then subjected to sequential immersion in the following liquids for 2 min each, in a fixed order: coffee (Nescafé^®^ Gold instant coffee; 4 teaspoons in 250 mL water; 50 °C), curry (Tommy^®^ curry sauce; 150 g sauce in 150 mL water; 50 °C), tea (Lipton^®^ Yellow Label Black Tea; 300 mL; pH 4; 50 °C), and alcohol (40%; room temperature). Between each staining medium, specimens were immersed in tap water at room temperature for 5 min. This sequential immersion protocol was chosen to reflect the additive and realistic nature of color challenges experienced by patients, rather than comparing each agent in isolation. For visual documentation, sample photographs can be accessed in the Supplementary Materials (Figure S1).

**Figure 1:**
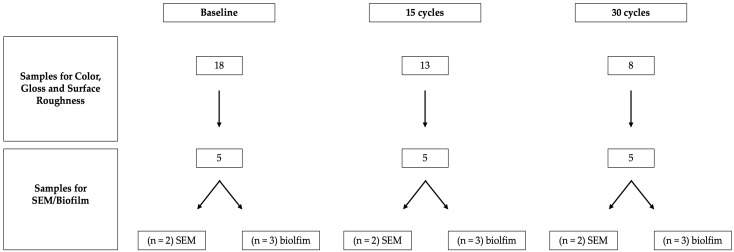
Schematic overview of specimen allocation and sequential removal during the accelerated aging protocol. At each time point (baseline, after 15 cycles, and after 30 cycles), five specimens per material were permanently removed for destructive analyses, including scanning electron microscopy (SEM; n = 2) and biofilm assessment (n = 3). The remaining specimens continued aging and were used for longitudinal analysis of color, gloss, and surface roughness.

Color, gloss, and surface roughness were re-assessed after 15 and 30 exposure cycles corresponding to a short-term, accelerated surface exposure model. At each time point (baseline, after 15 cycles, and after 30 cycles), five specimens per material were permanently removed for destructive analyses, comprising two specimens for SEM and three specimens for biofilm assessment. Due to the destructive nature of SEM and biofilm testing, these specimens were not returned to the aging protocol. The remaining specimens (n = 8) completed all 30 cycles and were included in the final statistical analysis of color, gloss, and roughness. A schematic overview of the sample allocation process is provided in Figure 1.

### 2.3. Color, Gloss and Surface Roughness Measurement

A spectrophotometer (CM-26dG, Konica Minolta, Inc., Tokyo, Japan) equipped with Spectra Magic NX software (v2.9, Konica Minolta Inc., Tokyo, Japan) was used to measure color and gloss. Discoloration was assessed in the CIELAB color space by recording L*, a*, and b* values (L* = lightness; a* = red–green axis; b* = yellow–blue axis). Overall color changes were calculated using the CIEDE2000 color-difference formula (ΔE00).

Surface roughness was measured using a contact profilometer with Precision Ultra software (Taylor Hobson^®^, AMETEK GmbH, Weiterstadt, Germany). Three measurements were taken per specimen at distinct locations spaced 2 mm apart and averaged to obtain the mean roughness value (Ra).

### 2.4. Scanning Electron Microscopy

For surface morphology assessment, specimens were ultrasonically cleaned prior to imaging. All specimens were then rinsed with distilled water, air-dried, mounted on SEM stubs, and sputter-coated with a 10 nm gold layer. Imaging was performed using a scanning electron microscope (GeminiSEM 450, Zeiss, Oberkochen, Germany) at an accelerating voltage of 10 kV, at magnifications of 500×, 2000×, and 5000×.

### 2.5. Microbial Adhesion

To assess microbial adhesion, a multispecies biofilm was cultivated on each specimen using six standardized supragingival species (*V. dispar*, *F. nucleatum*, *S. oralis*, *A. oris*, and *S. mutans*, *C. albicans*). Specimens were incubated for 20 h representing an early biofilm.

For culture preparation, each strain was transferred from Columbia blood agar (CBA; bioMatériaux SA, Marcy L’Étoile, France) into 3 mL modified fluid universal medium (mFUM) supplemented with 0.3% (*w*/*v*) glucose and pre-incubated at 37 °C, then stored at 4 °C until use. On day 2, a salivary pellicle was formed by incubating specimens for 4 h at room temperature in 4 mL processed saliva (1:1 dilution with 0.25% NaCl).

Biofilm growth comprised two phases. In phase 1, specimens were placed in 4 mL of medium containing 70% processed saliva (1:1 in 0.15% NaCl) and 30% mFUM with 0.3% glucose. In phase 2, the medium was supplemented with 0.15% glucose and 0.15% sucrose. A mixed inoculum was prepared by adjusting each culture to the same optical density and combining equal volumes of all six species. Pellicle-coated specimens were transferred into phase-1 wells, inoculated with 500 µL per well, and incubated anaerobically at 37 °C for 3 h. Specimens were then transferred to phase-2 wells and incubated for a further 17 h.

Biofilms were harvested by washing specimens three times in sterile 0.9% NaCl to remove non-adherent cells. Each specimen was placed in 500 µL 0.9% NaCl, vortexed for 2 min, shaken at 4 °C, and sonicated on ice using an ultrasonic wand, then stored at −20 °C. Serial 10-fold dilutions were plated on CBA in duplicate and incubated anaerobically at 37 °C for 3–4 days. Colony-forming units (CFU) were counted and expressed as CFU per specimen.

### 2.6. Statistical Analysis

Descriptive statistics (mean, SD; median, IQR) were calculated in Excel (v16.93; Microsoft, Redmond, WA, USA). Inferential statistics were performed using the online statistical software DATAtab (DATAtab e.U., Graz, Austria). Due to the small sample size (n = 8) and uncertain normality, non-parametric tests were applied. For comparisons between materials at a given time point, the Kruskal–Wallis test was used, followed by Dunn’s post hoc test with Bonferroni adjustment where appropriate. For within-material comparisons over time in the longitudinal dataset (color, gloss, and roughness; baseline and 15 and 30 cycles; n = 8), the Friedman test was applied with post hoc pairwise Wilcoxon signed-rank tests (Bonferroni-adjusted). Data are reported as median (IQR), and *p* < 0.05 was considered statistically significant.

For biofilm formation (log CFU), measurements were obtained from independent specimens at baseline, after 15 cycles, and after 30 cycles (n = 3 per material and time point). Within-material comparisons between time points were performed using pairwise Mann–Whitney U tests with Holm–Bonferroni adjustment for multiple testing. Between-material comparisons at each time point were conducted using the Kruskal–Wallis test (*p* < 0.05).

## 3. Results

### 3.1. Discoloration

Median L* (lightness) values showed a comparable pattern across materials, decreasing after 15 cycles and increasing again after 30 cycles, indicating an initial darkening followed by partial re-lightening with further exposure. At baseline and after 15 cycles, no statistically significant differences in L* were observed between Palapress^®^ and Valplast^®^. In contrast, Eldy Plus^®^ and Gingivamoll^®^ exhibited significantly higher L* values at both baseline and after 15 cycles. When changes over time were assessed within each material, all materials showed a significant decrease in L* after 15 cycles. After 30 cycles, L* values returned close to baseline levels for Palapress^®^ and Valplast^®^, whereas Eldy Plus^®^ and Gingivamoll^®^ showed a smaller recovery.

At baseline, all materials displayed positive a* values, with Gingivamoll^®^ showing the highest value, indicating a predominance of the red hue. For Palapress^®^, no statistically significant changes in a* were observed over time. In contrast, Valplast^®^ and Gingivamoll^®^ showed a decrease in a* over time, with a significant reduction from baseline to 30 cycles, indicating a gradual loss of the red component. Eldy Plus^®^ showed no significant change after 15 cycles; however, a decrease in a* was observed after 30 cycles.

Similarly, all materials exhibited positive b* values at baseline, again with the highest values for Gingivamoll^®^, indicating a predominance of the yellow component. After 15 cycles, b* increased significantly for all materials, reflecting a shift towards a stronger yellow hue. This change then stabilized, with no further significant increase from 15 to 30 cycles.

Regarding ΔE00, only Valplast^®^ showed significant changes over time and exhibited higher ΔE00 values compared with the other materials. Table 1 summarizes the median L*, a*, b*, and ΔE00 values and highlights statistically significant differences both between materials and within materials over time. Based on these findings, the null hypothesis regarding discoloration after accelerated aging was rejected.

### 3.2. Changes in Gloss and Surface Roughness

Compared with the other materials, Gingivamoll^®^ exhibited significantly lower baseline gloss (G). Across all materials, gloss showed a pattern similar to that observed for L*: a slight (non-significant) decrease after 15 cycles, followed by a significant increase at 30 cycles. Notably, at 30 cycles, all materials displayed significantly higher G values than at baseline (Table 2).

At baseline, surface roughness (Ra) was significantly higher for Gingivamoll^®^ than for the other materials. Overall, Ra increased over time for all materials, with significant increases evident by 30 cycles (and already after 15 cycles for Eldy Plus^®^), indicating progressive surface alteration with continued exposure (Table 2). Therefore, the null hypothesis for gloss and surface roughness was rejected.

### 3.3. Surface Morphology

**Figure 2:**
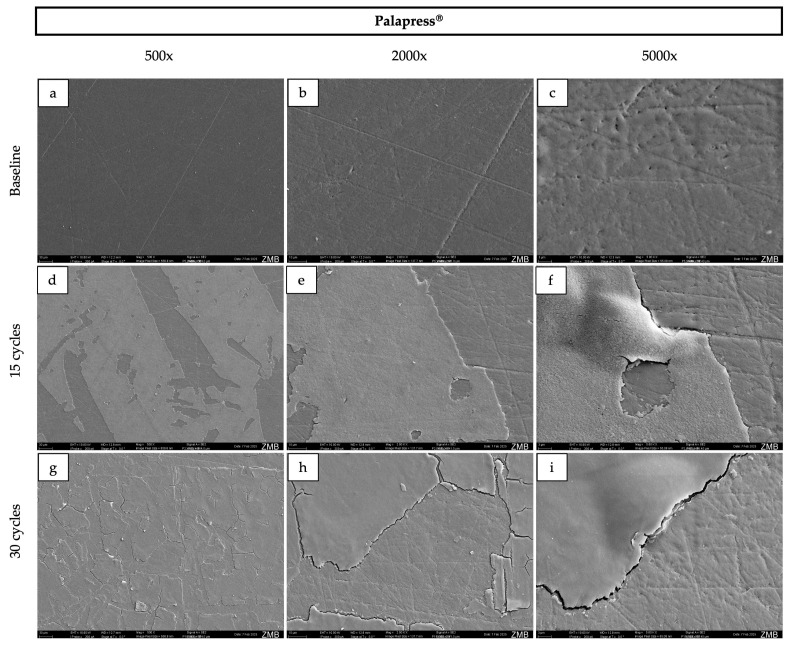
Representative scanning electron microscopy images of the surface of Palapress^®^ samples at baseline (**a**–**c**), after 15 cycles (**d**–**f**), and after 30 cycles (**g**–**i**) at 500×, 2000×, and 5000× magnification respectively. The corresponding field of view for each magnification is as follows: 500×, 570 × 410 µm; 2000×, 140 × 100 µm; and 5000×, 55 × 40 µm.

**Figure 3:**
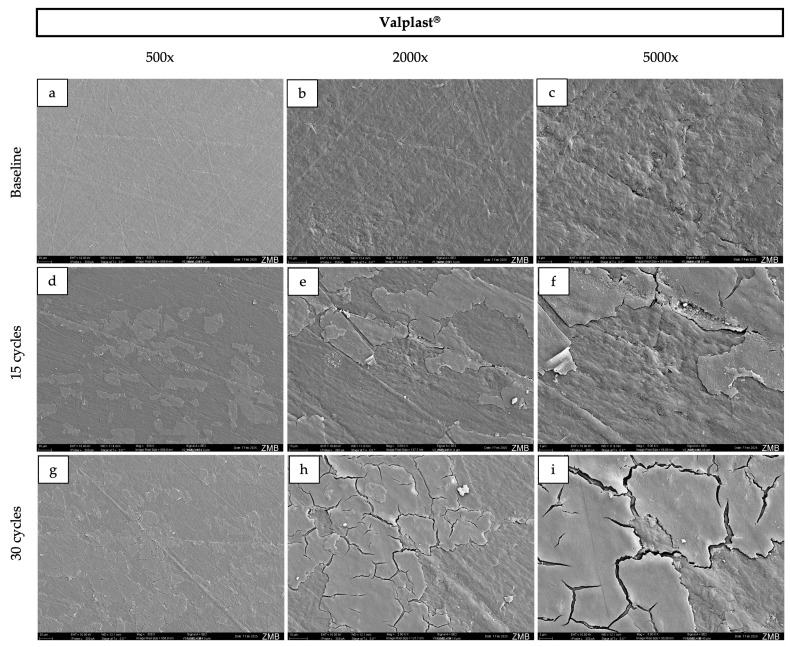
Representative scanning electron microscopy images of the surface of Valplast^®^ samples at baseline (**a**–**c**), after 15 cycles (**d**–**f**), and after 30 cycles (**g**–**i**) at 500×, 2000×, and 5000× magnification respectively. The corresponding field of view for each magnification is as follows: 500×, 570 × 410 µm; 2000×, 140 × 100 µm; and 5000×, 55 × 40 µm.

**Figure 4:**
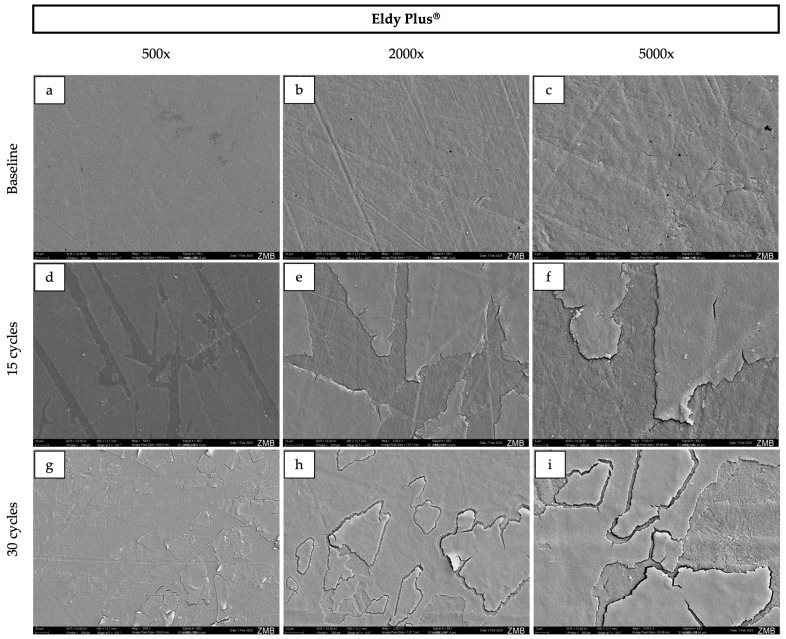
Representative scanning electron microscopy images of the surface of Eldy Plus^®^ samples at baseline (**a**–**c**), after 15 cycles (**d**–**f**), and after 30 cycles (**g**–**i**) at 500×, 2000×, and 5000× magnification respectively. The corresponding field of view for each magnification is as follows: 500×, 570 × 410 µm; 2000×, 140 × 100 µm; and 5000×, 55 × 40 µm.

**Figure 5:**
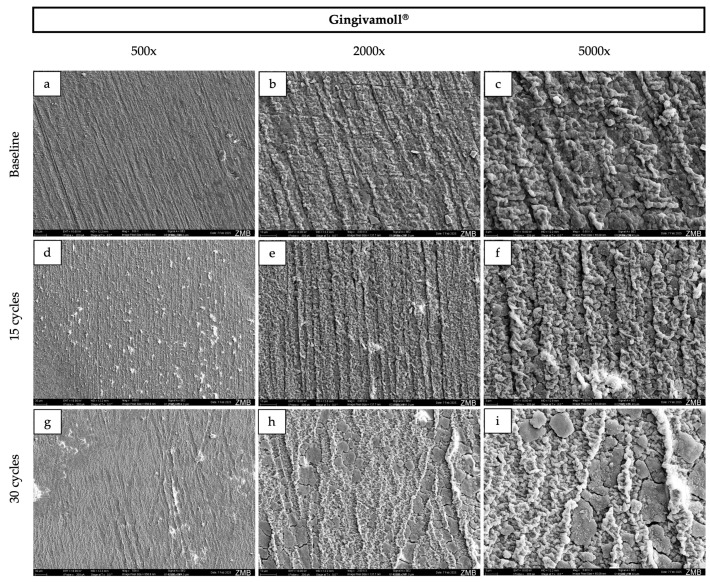
Representative scanning electron microscopy images of the surface of Gingivamoll^®^ samples at baseline (**a***–***c**), after 15 cycles (**d***–***f**), and after 30 cycles (**g***–***i**) at 500×, 2000×, and 5000× magnification respectively. The corresponding field of view for each magnification is as follows: 500×, 570 × 410 µm; 2000×, 140 × 100 µm; and 5000×, 55 × 40 µm.

At baseline, Palapress^®^, Valplast^®^, and Eldy Plus^®^ displayed comparable surface characteristics on SEM images (Figure 2, Figure 3, Figure 4 and Figure 5). The surfaces appeared relatively flat and smooth, with polishing-related grinding marks. In contrast, Gingivamoll^®^ (Figure 4) showed a rougher, more grooved surface texture with rounded, nodular features.

After 15 and 30 exposure cycles, all materials exhibited pronounced surface alterations. A smooth superficial coating developed on Palapress^®^, Valplast^®^, and Eldy Plus^®^, partially covering the original surface features. Conversely, Gingivamoll^®^ showed the formation of nodular agglomerates from 15 cycles onwards, which progressively filled the grooves rather than forming a continuous coating.

### 3.4. Microbial Adhesion

Median log CFU counts (Table 3) showed variability across aging cycles without a consistent directional trend. No statistically significant differences in biofilm formation were detected between time points within the same material (pairwise Mann–Whitney U tests with Holm–Bonferroni correction; *p* > 0.05). Similarly, no statistically significant differences were detected between materials at any time point (Kruskal–Wallis test, *p* > 0.05). Due to the limited sample size, the biofilm data are reported descriptively.

## 4. Discussion

This in vitro study compared four gingival epithesis materials under an accelerated staining protocol. All materials exhibited measurable changes in color, gloss, and surface roughness after exposure to curry, coffee, tea, and alcohol, and SEM revealed material-specific surface alterations. Accordingly, the null hypotheses for color stability and surface properties were rejected.

Color change in dental polymers has been widely reported and is driven by both chemical and mechanical factors, including pigment adsorption/absorption, surface defects, and polymer degradation [16,17,18,19,20]. Micro-cracks, porosities, and impurities may facilitate pigment uptake; consequently, rougher surfaces can show a higher discoloration potential [21,22]. In addition, hydrophilic surface components—particularly resin-based matrices—are often associated with more pronounced color changes than hydrophobic materials, especially when exposed to water-soluble colorants [23,24]. Residual monomers due to incomplete polymerization may further increase staining susceptibility in resin-based materials [25,26].

In the present study, all materials exceeded a ΔE00 value of 2.0, a commonly reported acceptability threshold for dental materials [27,28]. The greatest chromatic change was observed for the nylon-based material (Valplast^®^), which is in line with previous reports attributing the color instability of polyamides/co-polyamides to their hydrophilic behavior [26,29,30]. However, other studies have reported lower discoloration for nylon-based materials compared with acrylic resins [31]. Valplast^®^ is marketed as a modern polyamide with reduced water sorption relative to earlier generations; nonetheless, Nguyen et al. [32] demonstrated continuous water uptake and release of hydrophilic components (e.g., 12-ADL). Taken together, the present findings suggest that, despite advances in formulation, clinically relevant staining of Valplast^®^ remains likely under frequent exposure to common dietary colorants.

Interestingly, the silicone-based material showed the highest roughness values but did not exhibit the most pronounced discoloration. One explanation is its darker baseline appearance (high a* and b* values), which may reduce the visual impact of additional staining. This is consistent with Petropoulou et al. [33], who reported lower discoloration in gingiva-colored compared with tooth-colored materials. In addition, silicone materials are comparatively hydrophobic; because the present protocol relied largely on water-based staining media, interactions between hydrophilic pigments and a hydrophobic surface may have been limited, consistent with Lai et al. [30]. Overall, these observations indicate that surface roughness alone did not predict discoloration in this study, supporting the concept that color stability is multifactorial and material-dependent.

SEM findings further highlighted the distinct behavior of silicone compared with the harder polymer/resin materials. The hard materials presented smoother, readily polishable surfaces at baseline, whereas silicone exhibited grooves and a more irregular texture, likely reflecting the challenges of achieving a high-polish finish on elastomeric materials [3]. After staining, the hard materials developed a progressive superficial coating, while the silicone material showed nodular agglomeration that filled pre-existing micro-grooves rather than forming a uniform layer. Such surface layer formation is consistent with adsorption-based models of pigment deposition (e.g., multilayer adsorption concepts discussed for modern dental materials) [22]. This mechanism may also help explain the pattern of an initial decrease in lightness and gloss followed by a later increase: early cycles may promote pigment penetration and absorption into near-surface regions, darkening the surface and reducing specular reflection [34], whereas later cycles may promote accumulation of a superficial (surface) film that alters light reflection and increases measured gloss even when subsurface staining persists [35]. Additionally, all samples were immersed in 40% ethanol during each cycle, which can alter polymer surfaces by inducing swelling, extraction of low-molecular-weight components, and changes in surface energy. Such modifications may facilitate pigment infiltration, temporarily increase color differences (ΔE00), and influence surface roughness [36,37,38]. Over time, partial reorganization of the polymer network and solvent-mediated smoothing could contribute to the observed partial recovery in lightness and the increase in gloss. Ethanol may also enhance water uptake, further affecting the optical behavior.

Microbial adhesion was assessed descriptively using CFU counts. While rougher and more hydrophilic surfaces are often associated with increased microbial retention [39], biofilm formation has been reported to be less sensitive to roughness changes below approximately 0.2 µm [40]. In this study, roughness values for the hard materials remained well below this threshold, and the silicone material was close to it, which may partly explain the absence of consistent trends in CFU counts. Moreover, the observed superficial coating on hard materials and partial filling of grooves in silicone could reduce the availability of retention sites. Finally, although hydrophilic materials may facilitate initial attachment, bacterial binding can be weaker and more shear-sensitive, potentially increasing detachment during rinsing/handling steps [41]. Importantly, due to the small sample size (n = 3), these biofilm data are exploratory, providing a foundation for future studies with larger sample sizes (≥6–8 per group) to more reliably evaluate microbial adhesion.

To our knowledge, this is the first study to quantify multispecies biofilm formation on commonly used gingival epithesis materials in conjunction with prior SEM-based surface characterization. Clinically, the pronounced color instability of the nylon-based material suggests potential limitations for long-term esthetic indications, although material selection must also consider patient expectations, handling, cost, and availability.

Several limitations should be acknowledged. The in vitro protocol cannot fully reproduce the dynamic oral environment, including salivary flow, pellicle maturation, pH fluctuations, temperature changes, and mechanical wear (e.g., toothbrushing), all of which may influence staining and surface degradation. In addition, the number and intensity of exposure cycles were limited, and longer observation periods may yield different results. Finally, although standardized polishing improved comparability, the procedure may not reflect manufacturer-specific finishing protocols; therefore, the maximum achievable clinical performance of some materials may not have been fully captured. Further long-term in vivo studies are therefore warranted to validate these findings under clinically relevant conditions.

Although the accelerated protocol does not capture all intraoral variables, it enabled a standardized, head-to-head comparison of gingival epithesis materials under reproducible staining conditions. Overall, the results suggest that the primary clinical trade-off among materials lies in optical stability and surface integrity rather than in microbial adhesion under the conditions tested. Consequently, clinicians and technicians should primarily consider long-term color stability and polishability when selecting materials for gingival epitheses, which is reflected in the Section 5.

## 5. Conclusions

Within the limitations of this in vitro study, all tested gingival epithesis materials showed measurable changes in color and surface properties following accelerated aging in commonly consumed staining media. Changes were material-dependent, with the nylon-based material exhibiting the greatest color instability (ΔE00) and silicone the highest roughness (Ra). Hard materials (nylon, PMMA, and PETG-based polymer) developed a superficial coating, whereas nodular agglomerates formed on the soft silicone. Microbial adhesion was assessed descriptively and appeared similar among materials, offering pilot data that can guide future studies with larger sample sizes. Overall, the findings provide clinically relevant, comparative insight for material selection in gingival epitheses, and further long-term in vivo studies are warranted to confirm these observations under dynamic oral conditions.

## Figures and Tables

**Table 1:** L*, a*, b*, and ΔE00 values (median [IQR]) for each material at baseline, after 15 and 30 cycles. (n = 8 for each material).

L*									
**Material**		**Baseline**			**15 Cycles**			**30 Cycles**	
Palapress^®^	A	46.5 (0.6)	a	A	44.0 (1.6)	b	AB	46.7 (0.6)	a
Valplast^®^	A	46.1 (1.4)	a	A	43.6 (0.8)	b	A	45.5 (1.2)	a
Eldy Plus^®^	B	50.8 (0.4)	a	B	47.3 (1.0)	b	B	48.7 (0.8)	ab
Gingivamoll^®^	B	50.8 (1.5)	a	B	47.2 (1.0)	b	B	48.0 (0.7)	b
a*									
Palapress^®^	A	9.8 (0.7)	a	A	10.12 (0.6)	a	AB	9.99 (2.2)	a
Valplast^®^	AB	11.16 (2.8)	a	A	10.29 (2.9)	ab	A	8.18 (3.0)	b
Eldy Plus^®^	BC	14.16 (0.5)	a	AB	14.56 (0.6)	ac	BC	13.24 (0.7)	bc
Gingivamoll^®^	C	25.43 (1.5)	a	B	24.12 (1.5)	ac	C	23.70 (1.9)	bc
b*									
Palapress^®^	AB	5.46 (0.4)	a	A	8.34 (1.2)	b	AB	9.04 (1.1)	b
Valplast^®^	A	1.27 (0.8)	a	A	8.21 (1.3)	b	A	8.47 (1.4)	b
Eldy Plus^®^	BC	9.09 (0.5)	a	AB	12.7 (1.1)	b	BC	12.17 (0.5)	b
Gingivamoll^®^	C	12.84 (0. 3)	a	B	16.05 (0.9)	b	C	15.09 (0.7)	b
ΔE00									
Palapress^®^				A	3.95 (0.8)	a	A	2.98 (0.9)	a
Valplast^®^				B	6.29 (0.6)	a	B	7.60 (0.7)	b
Eldy Plus^®^				A	4.38 (1.4)	a	A	3.45 (0.8)	a
Gingivamoll^®^				A	4.18 (0.9)	a	A	3.77 (0.8)	a

Different capital letters represent significant differences between materials (*p* < 0.05; read vertically). Different lowercase letters represent significant changes over time within each material (*p* < 0.05; read horizontally). ΔE00 represents the color difference relative to baseline. Therefore, ΔE00 is not reported for the baseline time point.

**Table 2:** Gloss (G) and Roughness (Ra) values (median [IQR]) for each material at baseline, after 15 and 30 cycles. (n = 8 for each material).

G									
**Material**		**Baseline**			**15 Cycles**			**30 Cycles**	
Palapress^®^	AB	68.77 (2.2)	a	AB	53.24 (15.1)	a	A	91.32 (16.3)	b
Valplast^®^	AC	43.06 (7.8)	a	AC	40.56 (13.0)	a	BC	62.92 (6.8)	b
Eldy Plus^®^	B	70.56 (4.5)	a	B	65.94 (18.2)	a	AB	90.91 (8.3)	b
Gingivamoll^®^	C	2.24 (1.0)	a	C	1.8 (0.7)	a	C	3.43 (1.1)	b
Ra									
Palapress^®^	A	0.03 (0.00)	a	A	0.04 (0.02)	ab	A	0.05 (0.02)	b
Valplast^®^	AB	0.04 (0.01)	a	AB	0.05 (0.01)	ab	AB	0.06 (0.01)	b
Eldy Plus^®^	A	0.03 (0.02)	a	A	0.04 (0.01)	b	A	0.05 (0.01)	b
Gingivamoll^®^	B	0.16 (0.18)	a	B	0.2 (0.21)	a	B	0.21 (0.19)	b

Different capital letters represent significant differences between materials (*p* < 0.05; read vertically). Different lowercase letters represent significant changes over time within each material (*p* < 0.05; read horizontally).

**Table 3:** Biofilm formation measured at baseline, after 15 cycles, and after 30 cycles (n = 3 independent specimens per material and time point) expressed as median [IQR] values of logarithmic CFU counts (log_10_ CFU/specimen) for four denture base materials.

Biofilm									
Material		Baseline			15 Cycles			30 Cycles	
Palapress^®^	A	10.60 (9.19–11.38)	a	A	10.18 (8.98–10.98)	a	A	10.81 (9.31–11.25)	a
Valplast^®^	A	10.40 (8.90–11.25)	a	A	10.88 (9.52–11.31)	a	A	11.22 (9.69–11.63)	a
Eldy Plus^®^	A	12.23 (10.43–12.23)	a	A	11.83 (9.66–11.95)	a	A	10.48 (9.25–11.33)	a
Gingivamoll^®^	A	10.65 (9.45–11.07)	a	A	10.70 (9.38–10.85)	a	A	10.30 (9.30–10.65)	a

Different uppercase letters indicate statistically significant differences between materials at the same time point (Kruskal–Wallis test; *p* < 0.05). Different lowercase letters indicate statistically significant differences between time points within the same material (pairwise Mann–Whitney U tests with Holm–Bonferroni adjustment; *p* < 0.05). CFU = colony-forming units.

## Data Availability

The raw data supporting the conclusions of this article will be made available by the authors on request.

## Supplementary Materials

The following supporting information can be downloaded at: https://www.mdpi.com/article/10.3390/dj14030142/s1, Figure S1: representative macroscopic photographs of gingival epithesis material specimens. Columns represent materials (Eldy Plus^®^, Gingivamoll^®^, Valplast^®^, Palapress^®^), and rows represent time points (baseline, after 15 staining cycles, and after 30 staining cycles). Images are provided for visual documentation only; the samples depicted were not used in the experimental analyses.
