# Supplementary material for: In Vitro Comparison of Gingival Epithesis Materials: Color Stability, Surface Properties, and Microbial Adhesion After Staining
Source: Dent J (Basel). 2026 Mar 4;14(3):142. doi: 10.3390/dj14030142 (PMC13025953; doi:10.3390/dj14030142)
Supplement: Supplementary file 1 [file dentistry-14-00142-s001.zip › dentistry-4103127-supplementary.pdf]

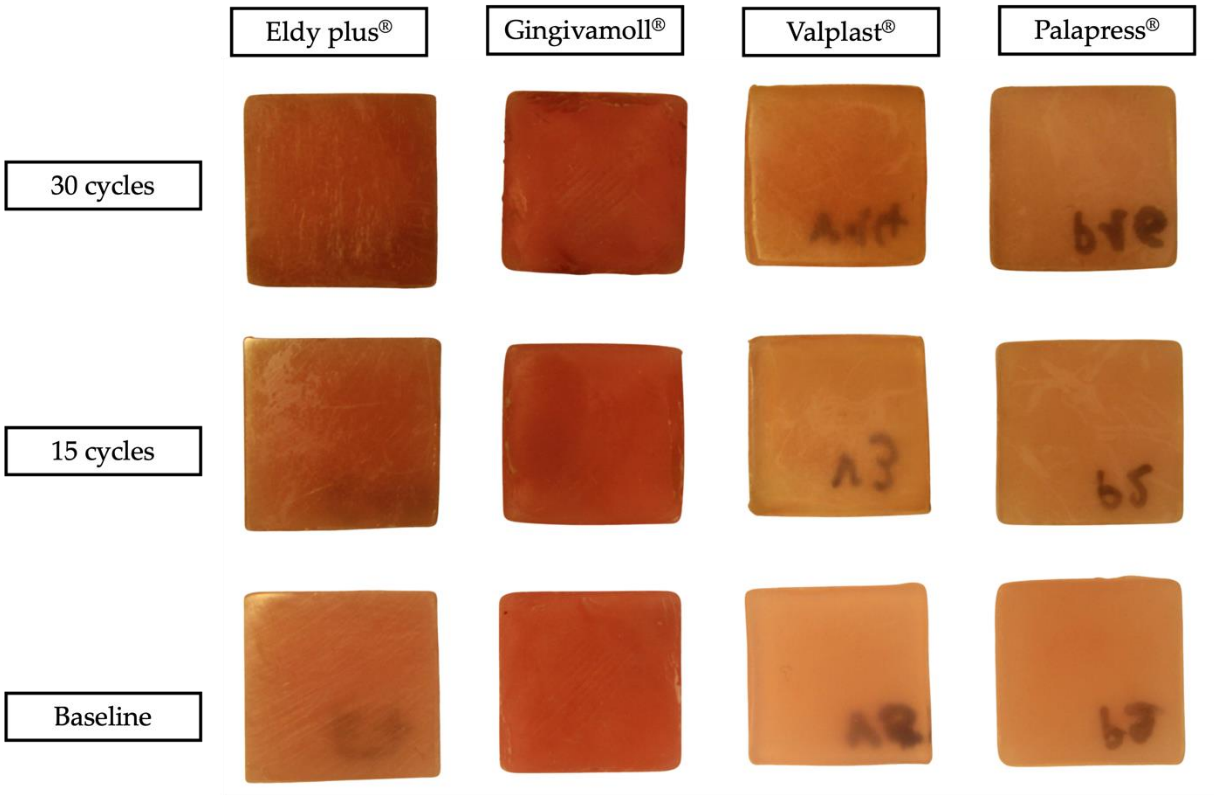

Figure S1. Representative macroscopic photographs of gingival epithesis material specimens. Columns represent materials (Eldy Plus®, Gingivamoll®, Valplast®, Palapress®), and rows represent time points (baseline, after 15 staining cycles, and after 30 staining cycles). Images are provided for visual documentation only; the samples depicted were not used in the experimental analyses.
